# On Surgical Dressings

**Published:** 1871

**Authors:** T. Hiron Bartleet

**Affiliations:** Surgeon to the Birmingham General Hospital


### *On Surgical Dressings.* By T. Hiron Bartleet, M.B., M.R.C.S., Surgeon to the Birmingham General Hospital.

I have been for many years increasingly disappointed with the ordinary dressings for suppurating wounds, such as amputations, excisions, burns, chronic abscesses, ulcers, etc.; and I believe a feeling of dissatisfaction is somewhat general. To this feeling may be attributed the great variety of dressings, and of modes of dressing, that have, from time to time, been recommended, and to it also the discarding by some of dressings altogether—the advocates of this plan recommending, for example, that a stump should be placed on a protected pillow and left altogether uncovered. Dressings have been, and still are, so frequently a harbor for dirt and decomposing discharges, that it is not surprising that their use should be less common and less complicated, or even dispensed with altogether.

Dressings usually have had some connection with lint. Now I have found lint to be, under almost all circumstances, a useless, and very often an injurious dressing. The lint of commerce is usually prepared from calico, instead of linen; and the cotton fibre is much more irritating than that of flax—a fact which will be readily understood by comparing the two under the microscope. Lint, too, is non-absorbent to a degree that few would expect, though every surgeon must have occasionally found the flaps of an amputation or open excised breast plugged by the lint laid over it, the first few drops of discharge having gummed down its edges to the parts near.

For the application of unguents, now so seldom thought necessary, linen is much more convenient than lint, as it receives the ointment more regularly, and is less heating and bulkly when applied.

I use lint myself rarely, except for two purposes : first, to check hæmorrhagic oozing, or to repress exuberant granulations, in which latter case sheet tin or lead is cleaner and more efficacious; and, secondly, for the application of lotions, where lint is useful for its capability of containing more of the fluid than linen will do—that is, when the lint is well soaked.

Even the plan of placing the suppurating part, as a stump, on a pillow covered with india-rubber sheeting, excellent though it is in its simplicity, has its inconveniences, from the tendency of the discharge to run off the pillow on to the bed-clothes. Moreover, this plan cannot be carried out in cases of suppurating wounds on the trunk.

It appears to me that what is wanted is, a dressing un-irritating, very absorbent, capable of retaining lotions and of being carbolised, and for general and especially for hospital use, of an inexpensive character and readily obtainable. I think all these requirements are found in picked oakum. In a former communication to *The Lancet* I mentioned that this dressing is in frequent use among American surgeons, and that it was, I believe, first introduced into this country by my friend, the late Mr. Redfern Davies. Oakum is readily to be obtained at gaols or workhouses in its crude state, in which condition it is used for caulking ships; and it is moderate in price. It consists of old ropes unpicked, and is therefore tow, but different from tow in two points : firstly, that it retains the agreeable and cleanly odour of tar, the presence of which may confer upon it some disinfecting properties; secondly, that, having been for some time twisted, when unpicked it retains a "springy" resilient condition, which renders it much more absorbent than tow. In a few words, picked oakum differs from tow just as curled horsehair differs from ordinary horsehair, and its use is in accordance with this difference.

I have been in the constant habit of using picked oakum for several years, first in the Birmingham Children's Hospital, and later at the General Hospital. I have used it in all kinds of suppurating wounds, and I believe invariably with advantage. It is some testimony to its value that every house-surgeon who has used it has expressed to me a high opinion of it.

It may, perhaps, appear unnecessary to write about the treatment of suppurating wounds when carbolic acid is doing so much to prevent their existence. But there are always cases in hospital that cannot be treated on Lister's plan, or in which that treatment has failed, or cases that have not come under treatment until suppuration has begun. Moreover, the time and care and extra assistance required in the sealing and after-dressing of wounds by Lister's process will prevent this treatment becoming very general.

Now, of course, I do not assert that picked oakum will heal an abscess, a sinus, or an ulcer; but when such are freely suppurating I think its use will materially expedite the cure by removing the pus as rapidly as it is formed, and I also believe that the comfort of the patient will be greatly increased by its use. In any hollow wound which cannot readily be placed in a position to drain itself, it is most useful. A few months ago I removed by the trephine a portion of the frontal and parietal bones of a child, whose skull had been fractured by a kick from a horse. The dura mater was uninjured. The hollow was a few days after constantly filled with pus, which, from its lying on the dura mater, gave me some anxiety. The application of a dossil of picked oakum maintained in place by a light bandage, and reapplied night and morning, kept the cavity quite empty.

I usually treat my amputations without dressings of any kind, only placing them on a protected pillow, and first putting under them a double handful of the oakum, which absorbs the secretions and prevents them running off the macintosh on to the bed. The oakum is readily removed two or three times daily, and, by its resiliency as well as by its cleanliness, is a comfort to the patient.

In abscess of the breast, both when the patient is in bed and when she is up, a double handful of oakum placed under and on each side of the breast, and kept in position either by a light bandage or by a piece of strapping, will keep the patient dry and clean far better than any other mode of dressing I know of. I am at present dressing a private case of excision of the breast in this way, and have used no other application from the very first. In fact, in any case of suppuration, in the use of drainage tubes, and especially in deep wounds, where it is difficult to place the opening of the wound in a depending position, the oakum will be found invaluable.

I know of only one objection to its use, and that is its apparent roughness. I say *apparent*, because I have heard no complaints of its being irritating to a patient. I used formerly, in special cases, to place between the wound and the oakum a piece of cap-net, which either did, or at all events appeared to do, all that was required. But now the oakum is being carded by machinery, and thus prepared it is much softer than the hand-picked, while it does not seem to have at all lost its springiness or its absorbent power. I am now using some sent to the General Hospital by

Messrs. Southall and Dymond, by whom it is also prepared, and I find this, which the manufacturers have named "Tenax," in every way a most useful form of oakum. I think it will probably take the place of hand-picked oakum, if it can be produced sufficiently cheaply. In hospital practice, or where the expense is an object, the tenax might be used for immediate contact with the wound, while a mass of the cheaper picked oakum might be placed outside this. The ordinary hand-picked oakum may be readily procured at a cheap rate at most gaols and workhouses.

I have found so much advantage to my patients and convenience to myself from the use of this dressing that I have felt constrained to write these few remarks, to induce others to participate in the advantages which are equally important to surgeon and patient.—*Lancet, May 13th.*

Birmingham, Jan. 1871.

